# Congenital Heart Disease Associated With Genetic Syndromes and Extracardiac Anomalies: A Six‐Year Epidemiological Study in a Brazilian Referral Center

**DOI:** 10.1002/bdr2.70111

**Published:** 2026-08-23

**Authors:** Letícia Cordeiro Rodriguez, Nicole Lerner, Maria de Fátima Monteiro Pereira Leite, Carla Verona Barreto Farias, Michail Barmpas, Dulce Helena Gonçalves Orofino, Juan Clinton Llerena Junior

**Affiliations:** ^1^ National Institute of Women, Children and Adolescents Health Fernandes Figueira/Oswaldo Cruz Foundation (IFF/FIOCRUZ) Rio de Janeiro Rio de Janeiro Brazil; ^2^ Federal University of the State of Rio de Janeiro (UNIRIO) Rio de Janeiro Rio de Janeiro Brazil

**Keywords:** congenital abnormalities, congenital heart defects, ECLAMC, epidemiology, perinatal care

## Abstract

**Background:**

Congenital heart diseases (CHDs) are structural and/or functional abnormalities of the heart that arise during embryonic cardiovascular development. They are the most common type of congenital defect and represent a major cause of neonatal morbidity and mortality, particularly when associated with genetic syndromes or other congenital anomalies. This study aims to analyze the clinical and epidemiological profile of CHD in newborns at a high fetal‐risk maternity hospital in Rio de Janeiro from 2018 to 2023, describing the types of CHD, associated anomalies and genetic syndromes, maternal risk factors, and neonatal outcomes.

**Methods:**

This is a descriptive, retrospective study using the database of the Latin American Collaborative Study of Congenital Malformations (ECLAMC) at Instituto Fernandes Figueira/Fiocruz (IFF/Fiocruz). Newborns with a confirmed diagnosis of CHD, born at IFF between January 2018 and December 2023 and properly registered in the ECLAMC program were included.

**Results:**

Among 5647 births, 225 newborns with CHD were identified and analyzed, corresponding to a prevalence of approximately 4%. The most frequent CHD was ventricular septal defect (VSD), present in 68 cases, followed by atrioventricular septal defect (AVSD) in 41 cases and coarctation of the aorta (CoA) in 27. Complex cardiopathies predominated, representing 60% of cases. Most diagnoses were established prenatally (93.3%), with a fetal echocardiogram accuracy of 78.5%. Associated congenital anomalies were identified in 64% of cases, predominantly affecting the gastrointestinal tract, central nervous system, and genitourinary tract. Genetic syndromes were confirmed in 95 patients (42.2%), with a predominance of Down syndrome (35), followed by Edwards syndrome (29) and Patau syndrome (10). The rate of hospital discharge alive was 53.3% in patients with isolated CHD, compared to 29% in those with associated syndromes or anomalies.

**Conclusions:**

IFF presents a highly complex patient profile, with a high prevalence of CHD associated with genetic syndromes and extracardiac anomalies, reflecting its role as a tertiary referral center. The presence of associated anomalies and syndromes negatively impacted neonatal prognosis, reinforcing the importance of prenatal diagnosis and specialized multidisciplinary care in the management of these conditions.

## 1 Introduction

Congenital heart diseases (CHDs) are abnormalities of cardiac structure and/or function that arise during embryonic development of the cardiovascular system. They constitute the most frequent group of birth defects, affecting approximately 1% of live births, and represent an important cause of neonatal and infant morbidity and mortality (Shabana et al. 2020; Zubrzycki et al. 2024). Globally, an estimated 1.35 million children are born each year with some form of CHD, with higher prevalence and worse prognosis in low‐ and middle‐income countries, where access to specialized diagnosis and treatment remains limited (Su et al. 2022).

More than 50 types of CHD have been described; among them, ventricular septal defect (VSD) is the most frequent, followed by atrial septal defect (ASD) and patent ductus arteriosus (PDA) (GBD 2017 Congenital Heart Disease Collaborators 2020). However, CHDs presenting coexistence of multiple structural defects overlap diagnostic groups, hindering their classification (Diz et al. 2021).

The gold‐standard examination for fetal diagnosis of congenital heart defects is fetal echocardiography, ideally performed between the 18th and 22nd weeks of gestation. However, it is a high‐cost test with limited availability, generally restricted to specialized centers, which precludes its routine use as a screening method. It is therefore recommended in the presence of risk factors or when abnormalities are identified on morphological ultrasound (Moon‐Grady et al. 2023; Pedra et al. 2019; Pinheiro et al. 2019).

In the postnatal period, transthoracic echocardiography is the main method for confirming or definitively excluding the presence of CHD, given its availability, low cost, and accuracy. It is indicated for patients with a prenatal diagnosis of cardiac abnormalities, clinical symptoms of CHD, or an abnormal pulse oximetry screening test (Plana et al. 2018).

Although they may occur in isolation, CHDs frequently coexist with genetic syndromes and extracardiac anomalies. Approximately 25% of cardiac defects are part of genetic syndromes, notably aneuploidies (Down, Edwards, Patau, and Turner syndromes), and microdeletions such as 22q11.2. Around 30% of newborns with CHD present concomitant extracardiac malformations, conditions that increase clinical complexity and impact prognosis (Chang et al. 2021; Diz et al. 2021; Dovjak et al. 2021; Pierpont et al. 2018).

In addition to genetic factors, the etiology of CHDs includes environmental factors (Meng et al. 2024). Among the main risk factors are maternal obesity and diabetes mellitus (Type 1, Type 2, or gestational), advanced maternal age, family history of CHD, and maternal infections during the first trimester (Gomersall et al. 2024; Khalilipalandi et al. 2024; Lemieux et al. 2024). Nevertheless, despite advances in genetic analysis techniques and studies on the environmental influences on cardiac development, genetic, or environmental causes are identified in fewer than half of cases (Meng et al. 2024; Wang et al. 2022).

In Brazil, data from the Live Birth Information System (SINASC) recorded 17,616 newborns with circulatory system malformations between 2018 and 2023, with a prevalence of 1.08 per 1000 live births (Ministério da Saúde, Departamento de Informática do SUS (DATASUS) 2024b). In addition, the Mortality Information System (SIM) showed an infant mortality rate (children under 1 year) of 1.11 per 1000 live births—which is higher than the number of cases diagnosed at birth, indicating underdiagnosis and underreporting in the country (Ministério da Saúde, Departamento de Informática do SUS (DATASUS) 2024a). This scenario reinforces the importance of institutional studies in reference centers, which can provide more accurate data and capture cases of higher complexity.

The National Institute of Women, Children and Adolescents Health Fernandes Figueira/Oswaldo Cruz Foundation (IFF/FIOCRUZ) is a national tertiary reference hospital for high‐risk fetal pregnancies, prenatal diagnosis of congenital malformations, and specialized neonatal care. This care profile concentrates highly complex cases, including newborns with multiple congenital anomalies and genetic syndromes, resulting in a population distinct from that usually described in population‐based studies worldwide.

The present study aimed to analyze the clinical and epidemiological profile of CHDs in newborns at IFF/FIOCRUZ between 2018 and 2023, describing CHD types, associated anomalies and genetic syndromes, maternal risk factors, and neonatal outcomes.

## 2 Methods

### 2.1 Study Design and Setting

This was an observational, descriptive, retrospective study conducted at Fernandes Figueira Institute/Fiocruz between January 2018 and December 2023.

### 2.2 Data Source

The study used data from the Latin American Collaborative Study of Congenital Malformations (ECLAMC) database, a monitoring program for newborns with congenital defects. ECLAMC records clinical and epidemiological data of newborns with at least one congenital defect, including defect description, clinical data, maternal history, prenatal examinations, obstetric, and family history.

### 2.3 Study Population and Inclusion Criteria

All newborns born at IFF between January 1, 2018 and December 31, 2023, with confirmed diagnosis of CHD by echocardiography and/or necropsy, and properly registered in the ECLAMC program, were included. No exclusion criteria were applied; all cases meeting the inclusion criteria were considered. Fields not completed in the ECLAMC forms were treated as missing data and were not imputed.

### 2.4 Variables

Maternal variables analyzed were: age, parity, multiple pregnancy, gestational complications, and family history of congenital malformation or genetic syndrome. Newborn variables analyzed were: sex, birth weight, gestational age, weight‐for‐gestational‐age adequacy, type of CHD, presence and type of associated congenital anomalies, presence and type of genetic syndromes, fetal echocardiogram accuracy, length of hospital stay, and hospital outcome.

### 2.5 Data Analysis

A descriptive analysis of the variables was performed, with categorical variables presented as absolute and relative frequencies and continuous variables as measures of central tendency. Analyses were performed in Microsoft Excel.

### 2.6 Ethics

The study was approved by the Research Ethics Committee of Instituto Fernandes Figueira (CEP/IFF) under approval numbers 4.148.310 and 7.440.329. All procedures performed in this study were in accordance with the ethical standards of the institutional research committee and with the 1964 Declaration of Helsinki and its later amendments. Informed consent was obtained from the legal guardians of all participants at the time of ECLAMC enrollment.

## 3 Results

Between January 2018 and December 2023, 5647 births occurred at IFF/Fiocruz. A total of 1152 cases of congenital malformation were identified, of which 838 were registered in ECLAMC (coverage of 72.7%). Of these, 225 had a confirmed diagnosis of CHD, corresponding to a prevalence of 4% at birth.

When classified by predominant defect, VSD was the most frequent CHD (32 cases), followed by cAVSD (29) and CoA (21). Considering all forms of presentation—isolated and combined—VSD was identified in 68 cases (30.2%), AVSD in 41 (18.2%), and CoA in 27 (12.0%). Complex CHDs predominated, representing 60% of the cases analyzed (Table 1).

**TABLE 1:** Distribution of congenital heart disease types.

CHD type	*N*
VSD	32
cAVSD	29
CoA	21
DORV	14
UV	14
ToF	13
HLHS	11
Heterotaxy syndromes	10
ASD	9
Vascular anomalies	8
ASD + VSD	6
TGA	6
PA	5
PS	5
Multiple	5
Dextrocardia	4
cAVSD + ToF	4
PA + VSD	3
TA	3
Double aortic arch	3
BAV	3
Ebstein anomaly	3
IAA	3
Ductus arteriosus abnormalities	2
HRHS	2
Polyvalvular dysplasia	1
AS	1
AS + OS	1
AS + MS + CoA	1
Truncus arteriosus type I	1
AVSD + TGA	1
Left ventricular aneurysm	1
TOTAL	225

*Note:* Cases were classified by the predominant CHD; combinations of multiple defects were grouped according to the lesion of greatest hemodynamic severity.

Abbreviations: AS, aortic stenosis; ASD, atrial septal defect; AVSD, atrioventricular septal defect; BAV, bicuspid aortic valve; cAVSD, complete atrioventricular septal defect; CoA, coarctation of the aorta; DORV, double outlet right ventricle; HLHS, hypoplastic left heart syndrome; HRHS, hypoplastic right heart syndrome; IAA, interrupted aortic arch; MS, mitral stenosis; PA, pulmonary atresia; PS, pulmonary stenosis; TA, tricuspid atresia; TGA, transposition of the great arteries; ToF, tetralogy of Fallot; UV, univentricular; VSD, ventricular septal defect.

### 3.1 Prenatal Diagnosis

CHD was diagnosed prenatally in 93.3% of cases. Seventy‐four percent of the pregnant women received complete prenatal care at IFF, while the remainder were referred from other centers due to diagnoses of congenital malformation. Fetal echocardiography was performed in 200 participants, with 187 cases with postnatal or necropsy confirmation. The prenatal diagnosis was accurate in 78.5% of cases (complete agreement); partially concordant in 14%, comprising absence of PDA and ASD, which cannot be confirmed during the fetal period, and predominantly false‐positive or negative findings for CoA; and discordant in 7%.

### 3.2 Newborn Characteristics and Associated Factors

Most patients were born at term (140/225; 62.2%); 80 (35.5%) were preterm, of whom approximately 20% were classified as extremely preterm (gestational age < 31 weeks). The stillbirth rate was 38.5% among preterm and 7% among term newborns. There was a slight predominance of males (112/225; 49.8%) over females (110/225; 48.9%), with two intersex cases (no karyotype available) and one case with missing data.

Regarding maternal factors, 36.4% of pregnant women were aged ≥ 35 years and 68% were multiparous, with a mean of 3.6 children. Diabetes mellitus was identified in 23/225 pregnancies (10.2%), TORCHS‐group infections in 23/225 (10.2%), and alcohol/drug use in 6/225 (3.3%). Family history of congenital malformation or genetic syndrome was reported in 42 pregnancies (18.7%), with 20 in first‐degree relatives.

The most prevalent gestational complications were urinary tract infection (32/225) and hypertensive disorders of pregnancy/pre‐eclampsia (25/225). However, since these conditions arise outside the period of embryogenesis, a direct correlation with the malformations could not be established. Clinical characteristics of newborns and their mothers are described in Table 2.

**TABLE 2:** Characteristics of newborns with congenital heart disease and their mothers.

Variable	Category	Live (*n*)	Stillborn (*n*)	Total *n* (%)
Sex	Female	88	22	110 (48.9)
	Male	90	22	112 (49.8)
	Intersex	0	2	2 (0.9)
	Missing data	—	—	1 (0.4)
Gestational age	≥ 37 weeks	130	10	140 (62.2)
	< 37 weeks	48	32	80 (35.5)
	Missing data	—	—	5 (2.3)
Weight for gestational age	AGA	104	21	125 (55.6)
	SGA	63	19	82 (36.4)
	LGA	11	1	12 (5.3)
	Missing data	—	—	6 (2.7)
Maternal age	< 35 years	119	21	140 (62.2)
	≥ 35 years	58	24	82 (36.4)
	Missing data	—	—	3 (1.4)
Parity	Nulliparous	56	13	69 (30.6)
	Multiparous	121	32	153 (68.0)
	Missing data	—	—	3 (1.4)
Twin pregnancy	Yes	10	0	10 (4.4)
	No	168	46	214 (95.1)
	Missing data	—	—	1 (0.4)
Family history of malformation/syndrome	Yes	31	11	42 (18.7)
	No	138	35	173 (76.9)
	Missing data	—	—	10 (4.4)
Gestational comorbidities	Alcohol/drugs	4	2	6 (3.3)
	Diabetes mellitus	17	6	23 (10.2)
	TORCHS	22	1	23 (10.2)
	Missing data	—	—	12 (5.3)
Confirmed genetic syndrome	Yes	69	26	95 (42.2)
	No	85	11	97 (43.1)
	Missing data	—	—	33 (14.7)
Associated malformations	Yes	105	39	144 (64.0)
	No	73	7	80 (35.6)
	Missing data	—	—	1 (0.4)
Total births		178	47	225 (100)

Abbreviations: AGA, appropriate for gestational age; DM, diabetes mellitus; GA, gestational age; LGA, large for gestational age; SGA, small for gestational age; TORCHS, toxoplasmosis, others, rubella, cytomegalovirus, herpes, and syphilis.

### 3.3 Associated Congenital Anomalies

**FIGURE 1:**
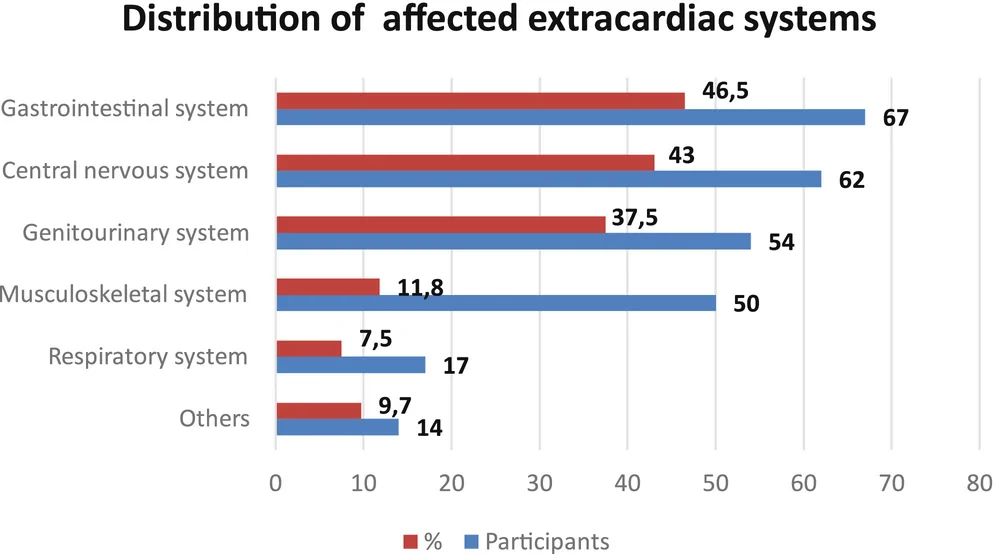
Distribution of extracardiac systems affected among the participants included in the study.

Associated extracardiac congenital anomalies were identified in 144/225 patients (64%), while 47/225 (20.8%) were born with isolated CHD. The most frequent anomalies affected the gastrointestinal tract (67/144; 46.5%), with predominance of abdominal wall defects (29/67), followed by central nervous system anomalies (62/144; 43%) and genitourinary tract anomalies (54/144; 37.5%) (Figure 1).

### 3.4 Associated Genetic Syndromes

**FIGURE 2:**
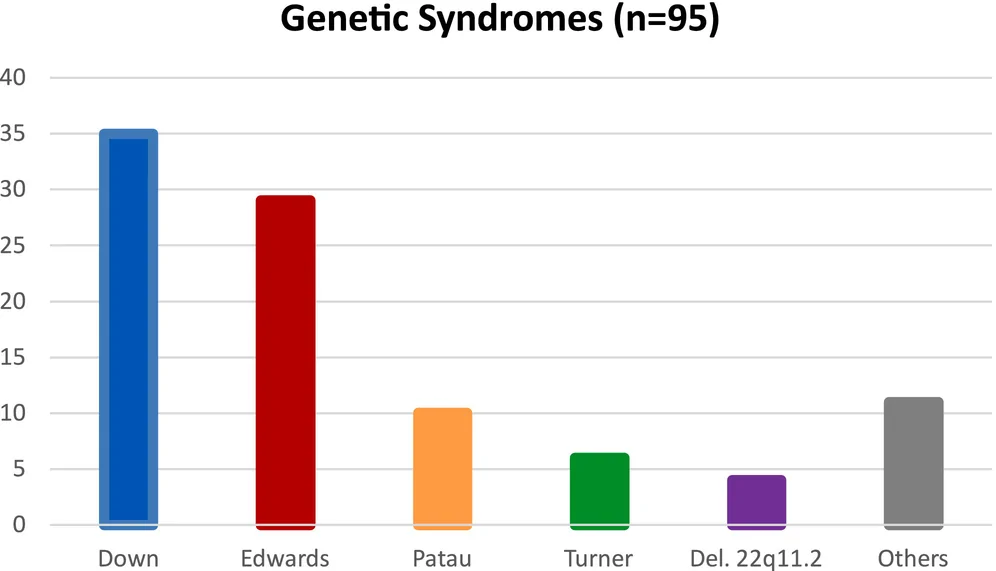
Distribution of types of syndromes confirmed by genetic study.

One hundred and thirty newborns (57.8%) had a syndromic phenotype, of whom 95 (73%) had a diagnosis confirmed by genetic testing—totaling 42.2% of the sample with a genetically confirmed syndrome. Aneuploidy syndromes (Down, *n* = 35; Edwards, *n* = 29; Patau, *n* = 10; Turner, *n* = 6) were confirmed by conventional karyotype. The 22q11.2 deletion syndrome (*n* = 4) was diagnosed by fluorescence in situ hybridization (FISH). Other syndromes were identified in 11 patients. The distribution of CHD types by genetic syndrome is shown in Figure 2 and Table 3.

**TABLE 3:** Distribution of congenital heart disease types by genetically confirmed syndrome.

Congenital heart diseases by genetic syndrome (*n* = 95)		
Syndrome	CHD type	*n* per defect
Down (*n* = 35)	cAVSD	19
	VSD	4
	cAVSD + ToF	3
	ASD	2
	CoA	2
	ToF	2
	PA	1
	PS	1
	TA	1
Edwards (*n* = 29)	VSD	12
	cAVSD	5
	DORV	2
	PA	2
	ASD + VSD	1
	CoA	1
	IAA	1
	Multiple	1
	Polyvalvular dysplasia	1
	TGA	1
	ToF	1
	UV	1
Patau (*n* = 10)	DORV	4
	CoA	1
	IAA	1
	Multiple	1
	UV	1
	VSD	1
	VSD + ASD	1
Turner (*n* = 6)	CoA	3
	BAV	2
	HLHS	1
22q11.2 deletion (*n* = 4)	ToF	2
	TA	1
	TGA	1
Other syndromes (*n* = 11)	ASD	3
	CoA	2
	ToF	2
	VSD	2
	ASD + VSD	1
	HLHS	1
Total		95

Abbreviations: ASD, atrial septal defect; BAV, bicuspid aortic valve; cAVSD, complete atrioventricular septal defect; CoA, coarctation of the aorta; DORV, double outlet right ventricle; HLHS, hypoplastic left heart syndrome; IAA, interrupted aortic arch; PA, pulmonary atresia; PS, pulmonary stenosis; TA, tricuspid atresia; TGA, transposition of the great arteries; ToF, tetralogy of Fallot; UV, univentricular; VSD, ventricular septal defect.

### 3.5 Prognosis and Hospital Outcomes

Of the 225 patients, 47 had isolated CHD, with a live birth rate of 89.3% (42/47), and 176 had CHD associated with genetic syndromes and/or other congenital malformations, with a live birth rate of 76.1% (134/176); two patients were not classified for this variable. The mean length of hospital stay was 15.7 days in the isolated CHD group and 28.9 days in the group with associated anomalies or syndromes.

**FIGURE 3:**
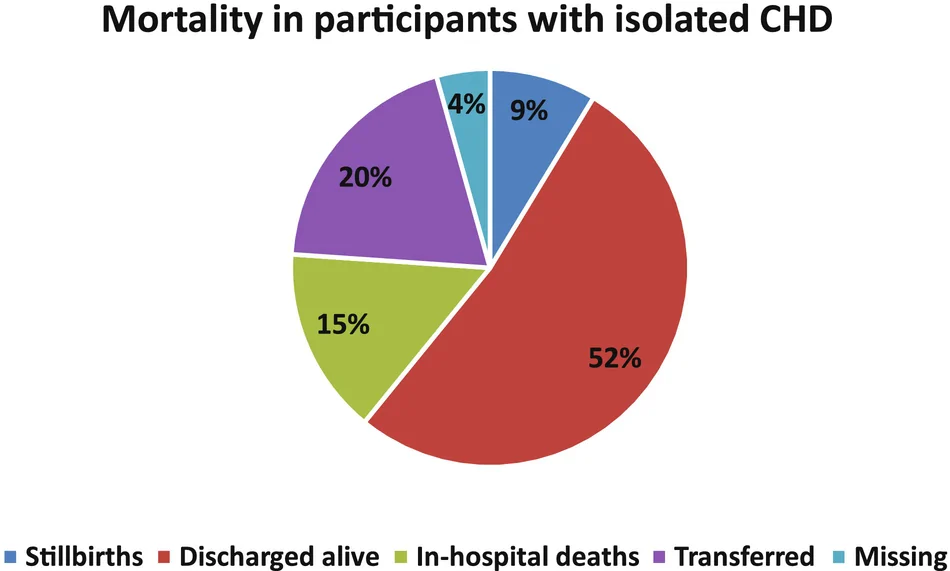
Prenatal and neonatal mortality in patients with isolated congenital heart disease.

**FIGURE 4:**
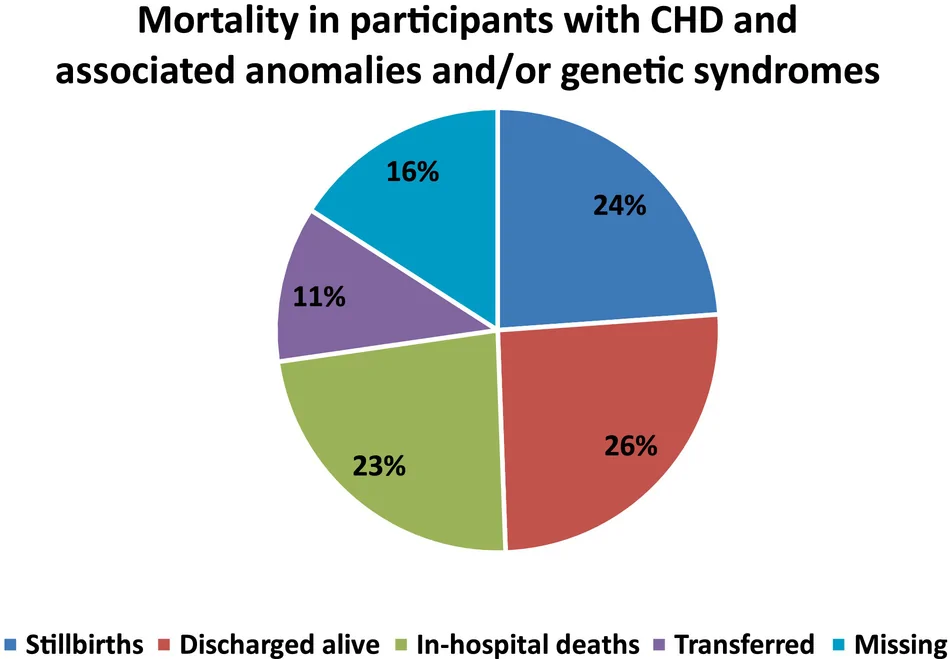
Prenatal and neonatal mortality in patients with congenital heart disease and associated congenital anomalies and/or genetic syndromes.

The rate of hospital discharge alive was 53.3% in the isolated group and 29% in the group with associated anomalies or syndromes. In‐hospital mortality among live births was 16.6% (7/42) in the isolated group and 30.5% (41/134) in the group with associated anomalies. Inter‐ward or inter‐hospital transfers occurred in 9/42 (21.4%) and 20/134 (14.9%) cases, respectively (Figures 3 and 4).

Among the stillbirths, only four cases were exclusively attributable to CHD, presenting heart failure at the time of fetal diagnosis: two patients with Ebstein anomaly and two with DORV, severe pulmonary stenosis, and right ventricular dysfunction. In the remaining cases, the diagnosed CHD did not present significant hemodynamic compromise, suggesting that associated syndromes or anomalies were determinants of the outcome.

## 4 Discussion

This study describes the clinical and epidemiological profile of 225 newborns with CHD attended at a tertiary reference maternity hospital in Rio de Janeiro over 6 years, representing a singular population characterized by high clinical complexity, predominance of complex CHDs, and marked coexistence of congenital anomalies and genetic syndromes.

The prevalence of CHD observed (4%) was markedly higher than that described in the general population (~1%) and in other ECLAMC participating centers. In the Latin American context, the overall malformation rate in ECLAMC between 1995 and 2008 was 2.7%, with Brazil presenting the highest rate among countries (4.2%) (Nazer and Cifuentes 2011). Among Brazilian centers of the network, ECLAMC‐PUCRS recorded an overall prevalence of 3.77%, while CHD‐specific studies reported prevalences of 1.1% at HC‐UFMG and 0.4% at Instituto Cândido Vargas (Paraíba) (Amorim et al. 2008; Araújo et al. 2014; Stefani et al. 2018). The overall prevalence of congenital malformations at IFF/FIOCRUZ during the study period (20.4%; 1152/5647) exceeds by more than sixfold the highest rate described among ECLAMC centers, resulting in a care profile not directly comparable to other services, even those participating in the same program.

The proportion of associated congenital anomalies (64%) is higher than reported in the literature (20%–30%) and reflects the systematic referral to IFF of pregnant women with prenatal diagnosis of multiple malformations, who benefit from the availability of specialized multidisciplinary support (Chang et al. 2021; Dovjak et al. 2021). In addition, the Brazilian legal context restricts pregnancy termination for fetal anomaly, in contrast to other countries where pregnancies with severe malformations frequently do not reach birth. This is a factor that contributes to the higher proportion of newborns with multiple anomalies (Amorim et al. 2008).

Regarding genetic syndromes, 73% of syndromic cases had cytogenetic and/or molecular genetic confirmation, with a predominance of aneuploidies, a finding consistent with previous studies, which identify chromosomal syndromes as the main identifiable cause of syndromic CHD. The profile of CHD by syndrome was consistent with expected patterns: predominance of cAVSD in Down syndrome; VSD and multiple complex CHDs in Edwards syndrome; complex conotruncal defects such as double outlet right ventricle in Patau syndrome; left‐sided obstructive defects in Turner syndrome; and tetralogy of Fallot and transposition of the great arteries in 22q11.2 deletion (Diz et al. 2021; Pierpont et al. 2018; Saliba et al. 2020).

Edwards syndrome (*n* = 29) showed a prevalence relatively close to that of Down syndrome (*n* = 35) in this sample; this differs from what is expected in the general population, where Down syndrome is considerably more frequent. It may be explained by the preferential referral of Edwards syndrome cases to IFF/FIOCRUZ, given the complexity of the support required for the multiple associated anomalies and predominance of complex combinations of defects (Chang et al. 2021; Diz et al. 2021; Meng et al. 2024).

The distribution of CHD types followed a pattern similar to that described in prior research: VSD as the most frequent CHD, followed by AVSD and CoA. However, the proportion of complex CHDs (60%) differed from that of usual population‐based samples, in which simple and isolated CHDs predominate (Saliba et al. 2020; Shabana et al. 2020).

In terms of sex distribution, a slight predominance of males was observed at an approximate ratio of 1.02:1. This finding is consistent with the literature, which describes a slight male predominance in CHD (De Oliveira et al. 2026; Diz et al. 2021). The high proportion of syndromic cases in the present sample may justify the balance between sexes, in contrast to the more pronounced male predominance described in populations with isolated CHD.

Concerning maternal risk factors, advanced age (≥ 35 years) was observed in 36.4% of pregnant women, an observation consistent with previous reports, demonstrating an association between advanced maternal age and increased risk of aneuploidies and congenital malformations (Gomersall et al. 2024; Khalilipalandi et al. 2024). Other classical risk factors, such as diabetes mellitus and family history, were observed in a minority of pregnant women, corroborating studies indicating that most CHD cases do not present an identifiable risk factor (Meng et al. 2024; Pierpont et al. 2018; Wang et al. 2022). It should be noted that IFF/FIOCRUZ is a reference hospital for fetal risk and not for maternal risk, which entails a lower proportion of pregnancies with maternal clinical risk factors compared to maternity hospitals for high obstetric risk.

Early diagnosis has been described as one of the main determinants of prognosis in newborns with CHD, allowing adequate perinatal planning with anticipated definition of the specialized team in the delivery room and early referral to the neonatal intensive care unit or to a cardiac surgery hospital, when indicated (Soares 2018; Su et al. 2022). The high rate of prenatal diagnosis observed (93.3%) and the accuracy of fetal echocardiography (78.5%, plus 14% partial matches) are strengths of the service and a direct reflection of the integrated work of fetal medicine with the other specialties. Cases with partial or inconclusive diagnoses correspond mainly to conditions recognized as difficult to assess in utero, such as coarctation of the aorta, or to findings that only manifest after the transition to extrauterine circulation, such as patent ductus arteriosus and patent foramen ovale.

A significantly more favorable prognosis was observed in patients with isolated CHD, a higher proportion of live births, shorter hospital stays, and a higher discharge rate compared with patients with genetic syndromes or multiple congenital anomalies, who had higher mortality and more prolonged hospitalizations. The high proportion of stillbirths (20.4% of the total), concentrated predominantly in the group with associated anomalies (89.4% of stillbirths), demonstrates that mortality is strongly linked to associated conditions and not only due to the hemodynamic severity of the CHD.

### 4.1 Limitations

Some limitations should be considered. First, ECLAMC registered 838 of the approximately 1152 malformation records of the period (coverage of 72.7%), due to limitations inherent to registry systems based on manual data collection, such as obtaining informed consent and occasional loss of records within the internal hospital workflow. In addition, some stillbirths may not have allowed sufficient time for the form to be completed, and some hospital discharges occurring on weekends may have escaped the registration routine.

Second, incomplete data were observed in some fields of the records, with occasional absence of information on specific variables. Moreover, patients transferred to other wards or hospitals had their follow‐up interrupted in the database, which may have underestimated late outcomes. However, there is no evidence that missing data differ systematically from the information included.

## 5 Conclusions

CHD is the most frequent group of structural birth defects and a major cause of neonatal morbidity and mortality, particularly when associated with extracardiac anomalies or genetic syndromes. This study characterized the profile of newborns with CHD at the Fernandes Figueira Institute, revealing a prevalence higher than that described in general centers, with predominance of complex CHDs, associated anomalies, and genetic syndromes.

Mortality was concentrated in the group with associated conditions, while patients with isolated CHD had a better prognosis, with higher rates of discharge alive and shorter hospital stays—a profile that reflects the role of IFF as a tertiary reference center offering specialized multidisciplinary support, neonatal intensive care, pediatric surgery, and genetics services.

The high rate of prenatal diagnosis observed, supported by the good accuracy of fetal echocardiography, was essential for perinatal planning and adequate referral to specialized management. These findings reinforce the importance of expanding access to fetal echocardiography and of training professionals to recognize CHDs and other malformations on routine morphological ultrasound, so that early detection reaches populations beyond major urban centers and contributes to reducing the access inequalities described in Brazil.

By providing data from a Brazilian tertiary referral center with a distinctive case‐mix, this study contributes to the epidemiological characterization of CHD at high‐complexity services and enables comparison with both national and international cohorts. These findings may also support future research on neonatal outcomes and on care strategies aimed at this clinically vulnerable population.

## Author Contributions

L.C.R. participated in the study conception, data collection and analysis, and manuscript writing. N.L., C.V.B.F., D.H.G.O., and M.B. participated in data collection and analysis of the manuscript. M.d.F.M.P.L. and J.C.L.J. participated in the study conception, supervision, and critical revision of the manuscript. All authors approved the final version.

## Funding

The authors have nothing to report.

## Ethics Statement

The ECLAMC project was approved by the Research Ethics Committee of Instituto Fernandes Figueira (CEP/IFF) under approval number 4.148.310 (CAAE: 59488716.1.1001.5269), with written informed consent obtained from the legal guardians of participants at the time of enrollment. The present study was approved by the same committee under approval number 7.440.329 (CAAE: 86297024.7.0000.5269). All procedures were conducted in accordance with the 1964 Declaration of Helsinki and its later amendments.

## Conflicts of Interest

The authors declare no conflicts of interest.

## Data Availability

The data that support the findings of this study are available on request from the corresponding author. The data are not publicly available due to privacy or ethical restrictions.
